# Seropositivity and diagnostic challenges of bovine brucellosis in Limpopo and Free State (2013–2022)

**DOI:** 10.4102/ojvr.v92i1.2224

**Published:** 2025-10-24

**Authors:** Emmanuel Seakamela, Itumeleng Matle, Sunday Ochai, Henriette van Heerden

**Affiliations:** 1 Department of Veterinary Tropical Diseases, Faculty of Veterinary Science, University of Pretoria, Pretoria, South Africa; 2 Department of Bacteriology, Onderstepoort Veterinary Research, Agricultural Research Council, Pretoria, South Africa; 3 Department of Agriculture and Animal Health, College of Agriculture and Environmental Sciences, University of South Africa, Pretoria, South Africa; 4 Antimicrobial Resource Unit, College of Health Sciences, University of KwaZulu-Natal, Durban, South Africa; 5 International Centre for Antimicrobial Resistance Solutions, Copenhagen, Denmark

**Keywords:** brucellosis, *B. abortus*, zoonosis, Limpopo, Free State

## Abstract

Brucellosis, a contagious zoonotic bacterial infection affecting livestock and wildlife, is primarily caused by *Brucella abortus*, globally. However, in South Africa, the true prevalence of bovine brucellosis remains unknown because of a lack of epidemiological data. Therefore, this study used diagnostic data to evaluate and determine the seropositivity of bovine brucellosis based on Rose Bengal test (RBT) screening and confirmation with complement fixation test (CFT) in Limpopo and Free State provinces between 2013 and 2022. The use and limitations of this data were also evaluated based on the bovine brucellosis scheme in South Africa. The study revealed the overall seropositivity of 4.2% (*n* = 8980/212 440) for bovine brucellosis based on RBT and CFT in series. In Limpopo province, the brucellosis seropositivity was slightly higher at 4.3% (*n* = 7488/173 011) compared to 3.8% (*n* = 1492/39 429) in Free State province. Analysis of brucellosis distribution over the study period indicated significant variation (*p* < 0.001) both between and within the provinces. Notably, the highest prevalence in Limpopo occurred during 2013–2017, whereas in Free State, peak prevalence was observed in 2013 and 2016. Challenges preventing an accurate reflection of the brucellosis seropositivity in these provinces for the period include a lack of data on vaccination history and herd status of the samples submitted, as well as the inability to match the CFT results from different laboratories, because some laboratories are only accredited to perform the RBT.

**Contribution:**

Insights gained from retrospective studies such as this study can play crucial roles in shaping effective control and preventative measures against bovine brucellosis. Given the challenges in obtaining confirmatory test results, we suggest that brucellosis tests be conducted at a single central laboratory or that the government provides a central database where all laboratories can enter their data. Furthermore, information submitted to the laboratories must make herd and vaccine history compulsory for sample submission to ensure more accurate data.

## Introduction

Brucellosis is a highly contagious zoonotic bacterial disease of humans, domestic animals and wildlife (WHO et al. 2006). This disease is caused by pathogenic members of the genus *Brucella*, which currently consists of 12 species (OIE 2022). Of the 12 species, *Brucella abortus, B. melitensis* and *B. suis* have the highest impact on domestic livestock productivity and human health, globally (Godfroid et al. 2011). *Brucella abortus* primarily infects cattle, while *B. melitensis* primarily affects sheep and goats and *B. suis* infects pigs (OIE 2022). In animals, brucellosis is acquired during parturition, when *Brucella*-infected birth fluids and aborted material contaminate the feed, vegetation and drinking water consumed by these animals (Acha & Szyfres 2001; FAO 2010). The disease in bulls may be sexually transmitted from infected females and occasionally leads to seminal vesiculitis, orchitis and testicular abscesses (Lopes, Nicolino & Haddad 2010; Moreno 2014; Tulu 2022). Once infected, animals do not show any clear signs of the disease until sexually matured females abort during the last trimester of pregnancy while males become infertile (WHO et al. 2006). Following abortions, animals may give birth to weak calves, stillbirths and produce less milk (Lopes et al. 2010). Other uncommon routes of infection including through cuts, inhalation, eyes and artificial insemination may occur (Crawford et al. 1990; Ko & Splitter 2003; Rankin 1965; Robinson 2003).

In South Africa, brucellosis is prevalent across the country, especially in intensive farming settings (Hesterberg et al. 2008). However, despite it being widespread in the country, the true burden of the disease remains unclear as most published data focused on specific areas and populations (Bishop 1984; Caine et al. 2017; Govindasamy et al. 2021; Hesterberg et al. 2008; Kolo et al. 2019; Marumo, Hlokwe & Kayoka-Kabongo 2023; McCrindle, Manoto & Harris 2020), while retrospective laboratory results (Kolo et al. 2021; Matle et al. 2021; Seanego et al. 2022) focused on only high-impact bovine population because of the bovine brucellosis scheme requirements. The scarcity of comprehensive studies on bovine brucellosis in South Africa, hinders the development of effective mitigation strategies.

Effective disease control programmes including those for brucellosis are developed based on a thorough understanding of the disease occurrence and the degree of infection in a particular area to ensure the reduction or eradication of the disease (Benkirane 2006; Dadar et al. 2021; Dean et al. 2012; Govindasamy et al. 2021; Hull & Schumaker 2018; Robinson 2003). In many developing countries, brucellosis schemes which include testing at herd level to ascertain animal health status are not practised because of shortage of financial and human resources, social stigmas such as discrimination of the infected herds and a lack of trust in government interventions (Franc et al. 2018; Hull & Schumaker 2018). For instance, in South Africa, animal brucellosis is a reportable and priority disease, controlled under the bovine brucellosis scheme (R.2483 of 09 December 1988) of the *Animal Diseases Act 35 of 1984*. The scheme prescribes the vaccination of all heifers aged 4-8-months with live attenuated *B. abortus* S19 vaccine, compulsory testing using Rose Bengal test (RBT) and confirmatory complement fixation test (CFT) with slaughter of seropositive animals, quarantine of farms and prohibiting movement of live animals from infected herds unless for slaughter. However, vaccination of heifers with S19 vaccine is mandatory with the bovine brucellosis scheme while testing is compulsory only for high-risk bovines (dairy and export) and voluntary for other animal owners (Department of Agriculture, Land Reform and Rural Development [DALRRD] 2016b). Vaccine and testing costs are only covered if done by a state veterinarian. The true prevalence of brucellosis in South Africa remains unknown, as the bovine brucellosis control programme is only partially implemented, with an increase in seroprevalence observed since the 1980s (Govindasamy 2020).

Diagnosis is based primarily on serological tests for practical purposes; however, confirmation is done with bacterial culture and polymerase chain reaction (PCR) methods (Matle et al. 2021). Culture is regarded as the gold standard and a definitive test for *Brucella* diagnosis, but it has certain limitations. It has a low sensitivity and may not always detect the bacteria in infected animals particularly from non-aborted samples and chronic cases (Navarro, Casao & Solera 2004). In addition, it is time-consuming, often requiring several days to weeks for results, and poses a significant risk of laboratory-acquired infection because of the highly infectious nature of *Brucella* spp (Hull & Schumaker 2018). These factors make culture less practical for routine diagnosis, especially in large-scale herd testing or in resource-limited settings.

Serological methods, such as RBT, CFT and indirect enzyme-linked immunosorbent assay (iELISA) are widely used around the world to detect brucellosis in livestock. However, these serological methods cannot distinguish between vaccine and wild strains of *Brucella*. In addition, samples of animals vaccinated after 8 months of age with S19 may produce cross-reacting antibodies, leading to false positive results (Simpson et al. 2018).

In this study, we focused on the epidemiology of brucellosis in Limpopo and Free State provinces, two regions characterised by significant livestock farming activities. According to a retrospective study done by Kolo et al. (2021), the seroprevalence of bovine brucellosis in these provinces between 2007 and 2015 was estimated at 13.5% in Free State and 19.7% in Limpopo. These figures underscore the endemic nature of the disease and highlight the regional variation in transmission dynamics, likely influenced by differences in animal movement, husbandry practices, vaccination coverage and diagnostic capacity.

Brucellosis, a highly contagious zoonotic disease, remains a significant challenge for livestock productivity and human health in South Africa, particularly in Limpopo and Free State provinces. Despite its widespread prevalence, the true burden of the disease is unclear because of the scarcity of comprehensive data, especially at the provincial level. Although the bovine brucellosis scheme is in place, it mandates only partial testing, primarily targeting high-risk cattle populations. Over time, financial constraints have shifted the focus from routine surveillance of high-risk populations to only focusing on infected herds, resulting in inadequate monitoring of the cattle population. Because of the focus on bovine brucellosis, we used retrospective laboratory results from 2013 to 2022 to assess the prevalence of bovine brucellosis in Limpopo and Free State provinces. Furthermore, we also assessed the retrospective data to inform recommendations aimed at reducing the prevalence of this disease and its control strategies in bovine populations.

## Research methods and design

### Study design

**FIGURE 1:**
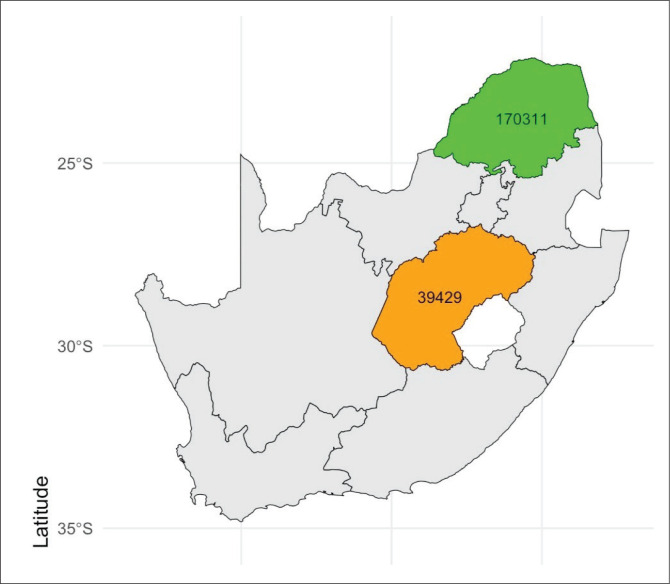
South Africa map showing Limpopo (1 710 311) and Free State (39 429) samples.

This study used a retrospective analysis of laboratory diagnostic data to assess the seropositivity of bovine brucellosis in Free State and Limpopo provinces, South Africa (Figure 1). This study relied on examining laboratory test records archived at Agricultural Research Council: Onderstepoort Veterinary Research (ARC: OVR) – Bacterial serology laboratory between 2013 and 2022.

### Study area

Limpopo province is situated in the northern part of South Africa and borders three countries: Botswana, Zimbabwe and Mozambique. It is also surrounded by Mpumalanga, Gauteng and North West provinces. The province is divided into five district municipalities, namely, Capricorn, Mopani, Waterberg, Vhembe and Greater Sekhukhune. Limpopo ranks fifth in South Africa in both surface area and population, covering an area of 125 754 km^2^ and being home to a population of 5 779 090. Three climatic zones are recognisable within the Limpopo province namely, subtropical lowveld region, subtropical plateau and an escarpment region (Limpopo Department of Agriculture 2008).

Free State province is in the geographical centre of South Africa, bordered by the Northern Cape, Eastern Cape, North West, Mpumalanga, KwaZulu-Natal and Gauteng provinces, as well as Lesotho. Although the Free State is the third largest (129 825 km^2^) province in South Africa, it has the second-smallest human population (2 834 714). Free State province is divided into one metropolitan municipality (Mangaung Metropolitan Municipality) and four district municipalities, which are further subdivided into 18 local municipalities.

### Data collection

Records of bovine serum samples received and assessed from January 2013 to December 2022 at ARC: OVR – Bacterial serology laboratory were used in this study. The serum samples were tested for brucellosis using the RBT and confirmed with the CFT, which typically considers a titre of ≥ 60 CFT international units (IU) and titre of ≥ 30 IU as positive in vaccinated and unvaccinated animals, respectively. Furthermore, titres between 18–24 IU and 30–49 IU are considered suspects in unvaccinated and vaccinated animals, respectively (DALRRD 2016a). Data abstraction was conducted by identifying complete and usable variables among available data set. Therefore, a dataset consisting of 212 440 bovine serum samples was compiled on the Microsoft 365 Excel spreadsheet. From the dataset, 173 011 samples were from Limpopo, while 39 429 were from Free State.

It is worth noting that in the past, the DALRRD approval was sufficient for government veterinary laboratories to test for controlled diseases, including brucellosis. However, the approval programme was terminated towards the end of 2014. Currently, it is mandatory, for a laboratory to obtain the South African National Accreditation System (SANAS) accreditation prior to testing of controlled diseases. Therefore, samples from unaccredited laboratories are forwarded to a national reference laboratory (Onderstepoort Veterinary Research [OVR], Pretoria) or other accredited laboratories for diagnosis. Some laboratories are accredited to perform either RBT alone or both RBT and CFT. Laboratories accredited only for the RBT are required to forward positive RBT samples to OVR or other accredited provincial laboratories for confirmation. Free State province has a veterinary laboratory accredited to test for RBT since 2021, while Limpopo province veterinary laboratories are not accredited to test brucellosis (https://nahf.co.za/dalrrd-approved-laboratories-2021-12-08/).

### Data analysis

We conducted descriptive statistical analysis to ascertain the number of positive cases and the percentage of animals that were positive for *Brucella*, with further stratification by location, year of detection and reactor animals. The statistical analysis was conducted in R Console, version 3.2.1, and a *p*-value of less than 0.05 was set as the benchmark for statistical significance.

### Ethical considerations

Ethical approval to conduct this study was obtained from the University of Pretoria, Animal Ethics Committee (REC 073-23). The permission to use the diagnostic results data was sought from the Agricultural Research Council (OVR).

## Results

A total of 212 440 samples were screened using RBT, of which 50 765 (23.9%) were positive and were subjected to CFT. Of the 50 765 CFT tested samples, 18% (*n* = 8980) were positive. Therefore, the overall seropositivity of bovine brucellosis in the current study based on RBT and CFT tests in series was 4.2% (*n* = 8980/212 440). There was a statistically significant (*p* < 0.001) difference in the seropositivity of brucellosis between the two provinces with 4.3% (*n* = 7488/173 011) and 3.8% (*n* = 1492/39 429) in Limpopo and Free State, respectively.

**FIGURE 2:**
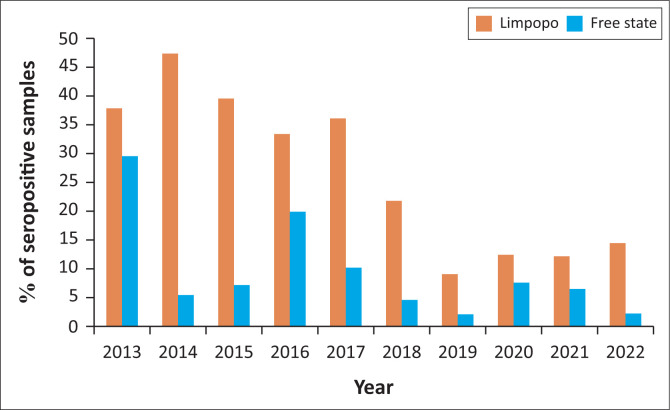
The distribution of seropositive cases by year of detection.

Figure 2 shows the distribution of seropositive cases by year. According to this figure, Limpopo province had statistically significantly higher seropositive cases than Free State province. The seropositivity of bovine brucellosis was highest in 2014 (47.2%), followed by 2015 (39.4%), 2013 (37.8%) and 2017 (36.1%) in Limpopo, while Free State had the highest proportions in 2013 (29.5%) and 2016 (19.9%) (Figure 2). This figure also shows that there was a significant decrease in the number of seropositive cases between 2018 and 2020 from both provinces. Furthermore, an increase in seropositivity has been observed from 2020 to 2022 in Limpopo, while a decline in cases has been observed in Free State during the same years.

**FIGURE 3:**
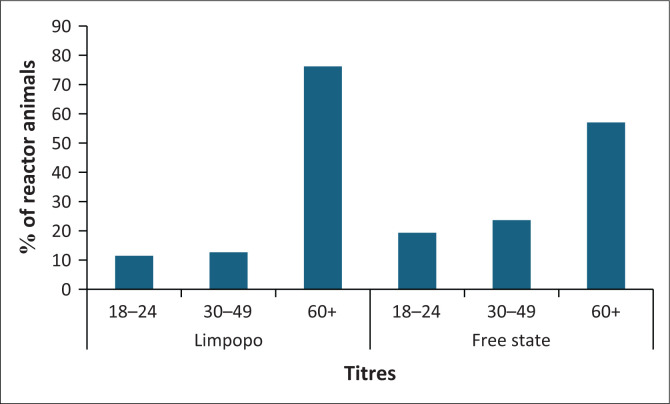
Distribution of complement fixation test titres by province (2013–2022).

The overall distribution of the reactor (seropositive) animals by province showed that Limpopo had a significantly higher number of cases (*n* = 7488) compared to Free State (*n* = 1921). In both provinces, the higher number of reactors was observed at the CFT titre > 60 IU/mL, followed by titres 30 IU/mL – 49 IU/mL and 18 IU/mL – 24 IU/mL. Notably, for titres 18 IU/mL – 24 IU/mL and 30 IU/mL – 49 IU/mL, Free State had a significantly higher prevalence of seropositive animals compared to Limpopo (*p* = 0.0472, Figure 3). In contrast, at the titre > 60 IU/mL, Limpopo showed a significantly higher seropositivity compared to Free State (*p* < 0.0001, Figure 3).

## Discussion

The current study used retrospective laboratory data from Limpopo and Free State provinces during 2013–2022 period and established the overall seropositivity of 4.2% for bovine brucellosis based on RBT and CFT results. Brucellosis is a socioeconomically important, zoonotic disease and is prevalent throughout the world. As a herd disease, its control relies on a thorough understanding of its epidemiology in different susceptible populations and areas. The retrospective analysis of laboratory data has contributed to the epidemiology of diseases in the absence of surveillance programmes. However, it is also worth noting that these studies may be limited, as the records used are of samples brought to the laboratory for routine testing. As mentioned, the bovine brucellosis scheme initially focused on partial testing, primarily targeting high-risk cattle populations. Over time, however, financial constraints have led to a shift in focus from routine surveillance of high-risk cattle populations to testing of infected herds, resulting in insufficient monitoring of the cattle population in South Africa. Therefore, they may not give a clear reflection on the prevalence of the disease. For instance, the seropositivity reported in the current study is based on individual animals. Furthermore, the information on the risk factors including age, sex, farm settings and management, husbandry, herds and vaccination was not recorded, which are beneficial to the better understanding of the dynamics of this disease. However, the trend that we observe is that seropositivity is increasing and varies between provinces. Therefore, the results of this study highlight the need for more comprehensive research studies for better understanding of this disease.

In the current study, the seropositivity by province was reported at 4.3% and 3.8% for Limpopo and Free State provinces, based on 173 011 and 39 429 cattle samples, respectively. The results of the current study align with those reported by Seanego et al. (2022) but are inconsistent with the results reported by Kolo et al. (2021). Most studies conducted in Limpopo, including Kolo et al. (2021), Seanego et al. (2022) and the current study, are retrospective in nature. Kolo et al. (2021) reported a 19.7% seropositivity rate based on 44 151 cattle samples tested between 2007 and 2015, which accounts for 4.16% of the total estimated cattle population in Limpopo (approximately 1 060 928 cattle), while Seanego et al. (2022) reported a seropositivity rate of 3.96% from samples tested between 2013 and 2016 with no indication of sample size. These studies, including the present one, provide valuable insights; however, their retrospective nature, the uncertainty regarding whether the same infected animals were resubmitted, the potential for large infected herds to skew the data and the limited sample sizes relative to the total cattle population all underscore the need for more comprehensive and up-to-date research.

In Free State, the retrospective study reported 13.5% seropositive rate of 17 138 samples tested between 2007 and 2015, which accounts for 0.74% of the total estimated 2 321 669 cattle population in the province (Kolo et al. 2021). A recent investigation concentrated on occupational human brucellosis reporting 10.7% from November 2019 to March 2020 associated with communal and commercial farms with brucellosis seropositivity of 1.1% in commercial herds and 8.7% in communal herds (van der Westhuizen et al. 2023). Another critical gap in the brucellosis control strategy is the lack of data on herd and vaccination history for submitted samples (Bishop 1984; Caine et al. 2017; Hesterberg et al. 2008; Kolo et al. 2021; Matle et al. 2021). This information is essential for evaluating the effectiveness of vaccination programmes and understanding the epidemiology of the disease. Without such data, it is difficult to assess the full impact of brucellosis on bovine populations in both provinces and across South Africa, leaving control measures inadequate and prevention strategies insufficiently informed.

Most brucellosis studies are cross-sectional, assessing the seroprevalence of the disease at a specific point in time within a particular population. Clinically, brucellosis is primarily associated with abortion, typically occurring during the first pregnancy following infection. Subsequent pregnancies often result in the birth of weak calves (Lopes et al. 2010). In a study by Mazwi et al. (2024), *Brucella* DNA, specifically *B. abortus* and/or *B. melitensis* was detected in 14.6% of cattle tissues (*n* = 41/280) from slaughtered animals at abattoirs in the Eastern Cape province. Interestingly, most animals that tested positive for *Brucella* by PCR were seronegative. The chronic nature of brucellosis means that cross-sectional studies often detect a low percentage of infected animals using serological tests, as the results are influenced by the timing of infection in each animal. Considering these findings, more comprehensive and longitudinal studies are needed to accurately assess the true prevalence and progression of brucellosis in cattle populations, as cross-sectional studies may underestimate the infection because of its chronic nature and variability in serological responses. However, as brucellosis is controlled by the bovine brucellosis scheme, these proposed studies are not allowed. According to the DALRRD disease report of 2020, a total of 1050 new *B. abortus* cases were reported between 2015 and 2019 in South Africa, which is three times less than what the current study observed (*n* = 4497) in the same years from the two provinces. Moreover, there has not been a notable effort in the country to control the disease. Although these figures confirm that brucellosis continues to spread in the country, the true burden of the disease remains speculative, and the goal to combat this disease seems unattainable. Previous studies have highlighted comprehensive and recent epidemiological knowledge as the principal tool for controlling the disease. However, despite the efforts to research this disease, the true prevalence in South Africa remains unknown. This is because of the discrepancies in disease reporting, record keeping and maintenance at the local, regional and national levels. For example, records at provincial laboratories are different from the records at the Agricultural Research Council, OVR as well as DALRRD reports. This may be attributed to the fact that currently, some laboratories are accredited to carry out diagnosis tests for controlled diseases including brucellosis. However, because of financial constraints and accreditation dynamics, some laboratories forward RBT-positive samples to the ARC-OVR and other provincial laboratories for confirmation. Because only positive samples are forwarded, it is difficult to establish the true prevalence of the disease. Therefore, the results of the current study highlight an urgent need to have a centralised reporting system so that all the basic epidemiology information can be channelled to one system and can be easily accessible. Moreover, the number of tests conducted and processed from Limpopo and Free State provinces is an important variable in economic studies that will be needed for continuing disease control efforts.

During the years under scrutiny, the seropositivity of brucellosis varied significantly between and within the provinces. In Limpopo, the seropositivity was high between 2013 and 2017, while cases in Free State fluctuated during the years with peaks in 2013 and 2016 only. Both provinces saw a huge decline between 2018 and 2020. The high prevalence recorded in the current study may be because of meteorological factors as well as farm management practices. Nyerere et al. (2020) modelled the impact of seasonal weather variations on infectiology of brucellosis and found that weather condition plays a significant role in the transmission rates of brucellosis. A study conducted in China on ‘spatiotemporal pattern evolution and driving factors of brucellosis’, reported that annual average temperature and precipitation play a major role (Xu & Deng 2022). The huge decline in cases between 2018 and 2020 may also be attributed to the coronavirus disease 2019 (COVID-19) pandemic which impeded the normal functionality of laboratories.

The seropositivity of brucellosis by reactor animals revealed that the overall prevalence was higher in Limpopo compared to Free State with titres exceeding 60 IU/mL being highest in both provinces. These results from Limpopo and Free State provinces, imply that bovine brucellosis is present in the cattle population tested. However, it is not possible to conclude the actual seroprevalence from the data because of the missing vaccination history of the animals tested. This can be explored further. To reach a better idea of the true seroprevalence, more comprehensive research needs to be conducted. Because brucellosis is a state-controlled disease, there is a need for change and implementation of stricter policies, a need for the private and government sectors to work together, and awareness campaigns.

## Conclusion

The study has estimated the seropositivity of brucellosis in Limpopo and Free State provinces. The information gathered from this study can be used to inform future epidemiological studies as well as control strategies. The study has also highlighted the gaps and limitations which may be used as a baseline for future research. The authors recommend that active surveillance and comprehensive studies be conducted to help improve our knowledge and understanding on the disease. The authors also recommend that awareness campaigns be conducted to assess and improve the knowledge and attitudes of farmers and the public. Because there are challenges in obtaining confirmation of test results at different laboratories in South Africa, we suggest that brucellosis tests be conducted at a single central laboratory or that the government provide a central database where all laboratories can enter their data.

### Limitations of the study

The study has used passive surveillance to assess the seropositivity of the brucellosis, which might be biased. The study does not have herd information which is a major limitation for a herd disease such as brucellosis. The data used in this study were based on RBT and CFT results which cannot differentiate between vaccine and wild strains, thereby making it difficult to project the true prevalence of brucellosis in the provinces. Factors such as age, sex, breed, seasonality, meteorological factors, and vaccination status of the animals were unavailable which could have played a significant role in the outcome of this study.
